# Population-specific factors associated with fractional excretion of uric acid

**DOI:** 10.1186/s13075-019-2016-6

**Published:** 2019-11-12

**Authors:** Ravi K. Narang, Zoe Vincent, Amanda Phipps-Green, Lisa K. Stamp, Tony R. Merriman, Nicola Dalbeth

**Affiliations:** 1 Department of Medicine, Faculty of Medical and Health Sciences, University of Auckland, 85 Park Road, Grafton, Auckland, 1023 New Zealand; 2 Department of Biochemistry, University of Otago, 710 Cumberland Street, Dunedin, 9012 New Zealand; 3 Department of Medicine, University of Otago, 2 Riccarton Avenue, Christchurch, 8140 New Zealand

**Keywords:** Gout, Urate, Uric acid, FEUA, Genetics

## Abstract

**Background:**

Reduced renal clearance of uric acid is a major contributor to hyperuricemia. The aim of this study was to examine clinical and genetic variables associated with fractional excretion of uric acid (FEUA).

**Methods:**

Participants (with and without gout) in the Genetics of Gout in Aotearoa study with available genotyping and FEUA data were included (*n* = 1713). Ten FEUA-associated loci detected within a genome-wide association study for serum urate in a European population were analysed. A polygenic score for FEUA was calculated in each ancestry group to model the cumulative effects of the genetic variants on FEUA. Associations between FEUA and both clinical variables and polygenic score were tested using linear regression models.

**Results:**

The mean (SD) FEUA was 5.13 (2.70) % in Eastern Polynesian participants, 4.70 (5.89) % in Western Polynesian participants, and 5.89 (2.73) % in New Zealand European participants. Although association with FEUA was observed for *SLC2A9* rs11942223 in New Zealand European participants (*P* = 2.39 × 10^− 8^), this association was not observed in Eastern or Western Polynesian participants. The polygenic score was positively associated with FEUA in all ancestry groups. In New Zealand European participants, body mass index, diuretic use, polygenic score, and male sex were associated with FEUA and explained 22% of FEUA variance in the regression model. In Eastern and Western Polynesian participants, the tested variables explained 10% and 4% of FEUA variance respectively.

**Conclusions:**

Both clinical and genetic variables contribute to renal clearance of uric acid*. SLC2A9* exerts effects on FEUA variance in people of European ancestry, but not in those of Polynesian ancestry. There is a large unexplained variance in FEUA, particularly in people of Polynesian ancestry.

## Background

Renal under-excretion of uric acid is the dominant cause of hyperuricaemia in 80–90% of people with gout [1, 2]. A number of clinical variables associate with the renal handling of uric acid, including body mass index (BMI) [3], kidney disease [4] and diuretic use [5]. These variables are also important contributors to the development of hyperuricaemia and gout.

Genetic variables also contribute strongly to renal uric acid excretion. Heritability for the fractional excretion of uric acid (FEUA) is estimated to be 46–96% [6–8]. In the Global Urate Genetics Consortium genome-wide association study (GWAS) for serum urate, Köttgen et al. [9] identified ten serum urate-associated single nucleotide polymorphisms (SNPs) that also associated with FEUA in a European sample. For all ten SNPs, the urate-raising allele was associated with a lower FEUA. Other SNPs have been identified that associate with serum urate and gout, and many of these SNPs encode transporters involved in renal uric acid excretion [9–13]. Genetic variants of these transporters can therefore potentially alter FEUA.

Population-specific differences in FEUA also exist and may contribute to differences in gout prevalence. In Aotearoa/New Zealand, there is a high prevalence of gout among Māori (indigenous New Zealanders) and Pacific peoples with 13.3% and 21.9% of men affected respectively, compared to 6.8% of non-Māori, non-Pacific men [14]. Renal under-excretion of uric acid contributes to hyperuricaemia in Māori and Pacific peoples [15, 16], and recent short-term intervention studies have shown that Māori and Pacific peoples have low FEUA responses to sugar-sweetened beverage [17] and frusemide challenges [18].

Currently, it is unclear which factors contribute the most to renal handling of uric acid and whether there are population-specific differences in the contribution of these factors. The aim of this study was to examine clinical and genetic factors associated with FEUA in an Aotearoa/New Zealand population.

## Methods

### Participants

Participants in the Genetics of Gout in Aotearoa study with available genotyping and FEUA data were included in this analysis. The study has been described in detail previously [12]. Study recruitment is ongoing and commenced in 2006. Participants were recruited from the Auckland, Waikato, Canterbury and Otago regions of Aotearoa/New Zealand. The analysis included participants with and without gout, defined by the 1977 American Rheumatism Association criteria for gout [19]. All participants completed a standardised study visit which included clinical assessment and collection of blood and urine for testing.

FEUA was calculated as ([urine urate × serum creatinine] / [serum urate × urine creatinine]) × 100. Urine analysis was performed on spot urine samples. Measurement of FEUA using spot urine samples has been previously demonstrated to be valid and reliable when compared to measurement of FEUA using 24-h urine samples [2]. Participants with a serum creatinine > 300 μmol/L (*n* = 12), or who were taking the uricosuric agents, probenecid (*n* = 44) or benzbromarone (*n* = 2), were excluded from the analysis. Participants taking febuxostat (*n* = 1) were also excluded from the analysis, as febuxostat use is reported to reduce FEUA [20]. Participants on allopurinol were not excluded, as allopurinol use does not alter FEUA [2, 20]. Participants were analysed according to self-reported ancestry: Eastern Polynesian (Māori and Cook Island), Western Polynesian (Samoa, Tonga, Niue, Tokelau, Tuvalu, Pukapuka), and New Zealand European. Māori and Pacific peoples were also analysed according to self-reported ethnicity, as recommended by the New Zealand Ministry of Health ethnicity reporting protocols [21]. In the case of multiple Polynesian ancestry including Māori (*n* = 36), participants were analysed as Eastern Polynesian or Māori. Ethical approval was granted by the New Zealand Multi-Region Ethics Committee (MEC/105/10/130) and all participants provided informed written consent.

### Single nucleotide polymorphism selection and genotyping

The ten SNPs associated with FEUA identified by Köttgen et al. [9] were genotyped using a combination of Taqman SNP genotyping on a Roche LightCycler 480 and the Sequenom MassARRAY System, as previously described in [12], and a CoreExome array (Illumina). At *SLC22A12/NRXN2*, SNP rs3825018 (surrogate for rs505802 [10]) was genotyped, and the previously reported rs11942223 was genotyped at *SLC2A9*. At *SLC22A11* (rs2078267), the opposite strand to the allele reported by Köttgen et al. [9] was genotyped using Sequenom/Taqman. Four SNPs were genotyped using both Sequenom/Taqman and a CoreExome Gene-Chip methods (*ABCG2* rs2231142, *SLC2A11* rs2078267, *RREB1* rs675209 and *UBE2Q2* rs1394125). There was a > 99% concordance in genotype for each SNP, and as such, any overlapping genotypes were combined. *GCKR* rs1260326 was genotyped using the CoreExome array alone. The remaining SNPs were genotyped using Sequenom/Taqman alone. The effect allele for each SNP associated with FEUA was defined by Köttgen et al. [9].

### Polygenic score

For each ancestry and ethnicity group, a weighted polygenic score for FEUA was calculated to model the cumulative effects of the genetic variants on FEUA. Genetic variants for *HLF*, *NFAT5* and *IGFR1* were not included in polygenic score modelling due to a high proportion of missing genotyping data for these loci. For each of the FEUA-associated SNPs, effect sizes (*β*) for FEUA association within each ancestry and ethnicity group from this analysis were used for polygenic score modelling. For each individual, these effect sizes were multiplied by the number of effect alleles present and summed into a weighted polygenic score for FEUA. A higher polygenic score indicates a greater genetic predisposition for a higher FEUA.

### Study power

Power calculations were performed using Quanto (version 1.2.4, May 2009, University of Southern California). Power calculations were performed a priori for the four loci encoding urate transporters (*ABCG2* [encoding ABCG2], *SLC22A11* [encoding OAT4], *SLC22A12* [encoding URAT1], and *SLC2A9* [encoding GLUT9]). The power to detect association with FEUA was determined at *P* < 0.005 significance for a range of presumed effect sizes (0.2–1.0%). For Eastern Polynesian participants, power to detect an association with FEUA was > 80% for *SLC22A11* at an effect size of 1.0%, and for *SLC22A12* and *SLC2A9* at an effect size ≥ 0.8% (Additional file 1: Table S1). For Western Polynesian participants, power to detect an association with FEUA was > 80% for *ABCG2* and *SLC22A12* at an effect size ≥ 1.0%. For New Zealand European participants, power was > 80% for all four SNPs at an effect size ≥ 0.6% (Additional file 1: Table S1). Power calculations for participants analysed according to self-reported ethnicity are shown in Additional file 1: Table S2.

We also performed post hoc power calculations for all ten FEUA-associated SNPs at *P* < 0.005 significance using association data generated from this analysis (Additional file 1: Tables S3 and S4). Power to detect association with FEUA at > 80% was only present for *SLC2A9* in New Zealand Europeans.

### Statistical analysis

Data were analysed using IBM SPSS Statistics 25. Clinical characteristics were summarised using standard descriptive statistics including means, standard deviations (SD), number and percent. Association of FEUA with categorical clinical variables (sex, BMI categories, current alcohol intake, diuretic use and estimated glomerular filtration (eGFR) categories) and continuous clinical variables (age, serum triglyceride, serum low-density lipoprotein-cholesterol (LDL-C)) were tested in a linear regression model with FEUA as the dependent outcome. For the linear regression analysis, serum triglyceride was log-transformed due to a right-skewed distribution in all ancestry groups. Linear regression of the ten SNPs (according to the number of effect alleles) with FEUA as the dependent variable was performed with adjustment for age and sex. Association of the polygenic score with FEUA was also tested using linear regression. All clinical variables and the polygenic score were entered into a linear regression model with FEUA as the dependent variable. The adjusted *R*-square was used to estimate the proportion of the variance in FEUA explained by the model. In the analysis for Eastern Polynesian, Western Polynesian, Māori and Pacific participants, the linear regression analysis was also adjusted for a measure of Polynesian ancestry, based on the number of Polynesian grandparents. Variables associated with FEUA were also tested for their association with individual components of the FEUA calculation (serum urate, serum creatinine and urinary urate/urinary creatinine) in a univariate linear regression analysis and a multivariable linear regression analysis including all clinical variables and the polygenic score. Data from regression analyses were summarised using unstandardised *β* coefficients and standardised *β* coefficients. The standardised *β* coefficient refers to the number of standard deviations the dependent outcome would change, per standard deviation increase in the tested variable. Where multiple testing was used in the SNP analysis, experiment-wide significance was defined as *P* < 0.005.

## Results

### Clinical features of study participants

The clinical characteristics are shown in Table 1. There were 1713 participants in total. There were 483 (28.2%) Eastern Polynesian participants, 282 (16.5%) Western Polynesian participants and 948 (55.3%) New Zealand European participants.

**Table 1:** Clinical characteristics of the study participants

	All *n* = 1713	Eastern Polynesian *n* = 483	Western Polynesian *n* = 282	New Zealand European *n* = 948
Age, mean (SD), years	54.1 (17.5)	50.0 (17.1)	43.7 (16.5)	59.4 (15.9)
Male sex, *n* (%)	1227 (71.6)	286 (59.2)	179 (63.5)	762 (80.4)
BMI, mean (SD), kg/m^2^	30.8 (6.8)	32.6 (7.7)	34.0 (6.5)	28.9 (5.7)
< 25 kg/m^2^, *n* (%)	325 (19.0)	65 (13.5)	22 (7.8)	238 (25.1)
≥ 25 kg/m^2^ and < 30 kg/m^2^, *n* (%)	549 (32.0)	122 (25.3)	50 (17.7)	377 (39.8)
≥ 30 kg/m^2^, *n* (%)	839 (49.0)	296 (61.3)	210 (74.5)	333 (35.1)
Current alcohol intake, *n* (%)	1002 (58.5)	208 (43.1%)	101 (35.8)	693 (73.1)
Gout, *n* (%)	896 (52.3)	206 (42.7)	124 (44.0)	566 (59.7)
Allopurinol use, *n* (%)	709 (41.4)	170 (35.2)	109 (38.7)	430 (45.4)
Diuretic use, *n* (%)	322 (18.8)	93 (19.3)	38 (13.5)	191 (20.1)
Serum creatinine, mean (SD), μmol/L	101.1 (32.7)	95.9 (29.1)	100.9 (33.5)	103.7 (33.9)
eGFR ≥ 60 mL/min/1.73 m^2^, *n* (%)	1274 (74.4)	380 (78.8)	213 (76.6)	657 (71.4)
Serum urate, mean (SD), mmol/L	0.39 (0.11)	0.38 (0.10)	0.41 (0.11)	0.38 (0.10)
Serum LDL-C, mean (SD), mmol/L	2.84 (0.96)	2.82 (0.96)	2.8 (0.92)	2.88 (0.97)
Serum triglyceride, mean (SD), mmol/L	2.06 (1.43)	2.12 (1.44)	1.97 (1.31)	2.06 (1.45)
FEUA, mean (SD), %	5.48 (2.71)	5.13 (2.70)	4.70 (5.89)	5.89 (2.73)

*BMI* body mass index, *eGFR* estimated glomerular filtration rate, *FEUA* fractional excretion of uric acid, *LDL-C* low-density lipoprotein-cholesterol, *SD* standard deviation

The frequency distribution of FEUA in the entire study population and according to ancestry group is shown in Additional file 1: Figure S1. The mean (SD) FEUA of the entire study population was 5.48 (2.71) %. The mean (SD) FEUA was 5.13 (2.70) % in Eastern Polynesian participants, 4.70 (5.89) % in Western Polynesian participants and 5.89 (2.73) % in New Zealand European participants (ANOVA *P* = 2.10 × 10^− 12^). There was no significant difference in FEUA between Eastern and Western Polynesian participants (Tukey post hoc *P* = 0.08). FEUA was significantly lower in Eastern and Western Polynesian participants, compared to New Zealand European participants (Tukey post hoc *P* = 1.18 × 10^− 6^ and *P* = 5.30 × 10^− 9^ respectively).

### Association of FEUA with clinical variables in linear regression analysis

In the linear regression analysis, male sex, BMI ≥ 30 kg/^2^, and diuretic use were inversely associated with FEUA in Eastern Polynesian and New Zealand European participants (Table 2). In Western Polynesian participants, only male sex was inversely associated with FEUA; however, inverse association of BMI ≥ 30 kg/m^2^ and diuretic use with FEUA was approaching statistical significance (*P* = 0.07 and *P* = 0.06 respectively). The remaining clinical variables (current alcohol intake, eGFR < 60 ml/min, age, serum LDL-C, and serum triglyceride) were not associated with FEUA in any ancestry group (Table 2).

**Table 2:** Linear regression analysis of clinical variables with fractional excretion of uric acid

	Eastern Polynesian *n* = 483				Western Polynesian *n* = 282				New Zealand European *n* = 948			
	*β*	SE *β*	Standardised *β*	*P*	*β*	SE *β*	Standardised *β*	*P*	*β*	SE *β*	Standardised *β*	*P*
Male sex	− 0.52	0.25	− 0.10	0.04	−0.67	0.32	− 0.13	0.04	−1.72	0.22	−0.25	4.73 × 10^−15^
BMI ≥ 30 kg/m^2^	− 0.78	0.18	− 0.21	2.16 × 10^−5^	− 0.49	0.27	− 0.12	0.07	−0.82	0.12	−0.23	4.77 × 10^− 12^
Current alcohol intake	0.35	0.25	0.06	0.17	−0.37	0.31	−0.07	0.23	−0.35	0.20	−0.06	0.08
Diuretic use	−0.85	0.34	−0.12	0.01	−1.01	0.54	−0.14	0.06	−0.87	0.23	−0.13	2.11 × 10^−4^
eGFR < 60 ml/min	− 0.03	0.34	−4.05 × 10^− 3^	0.94	− 0.25	0.44	− 0.04	0.57	0.36	0.21	0.06	0.10
Age	−0.01	0.01	−0.08	0.13	2.53 × 10^− 3^	0.01	−0.02	0.82	−0.01	0.01	−0.05	0.16
LDL-C	0.06	0.13	0.02	0.63	0.04	0.17	0.02	0.81	1.66 × 10^−3^	0.09	0.00	0.98
Triglyceride	−0.03	0.15	−0.01	0.83	−0.07	0.18	−0.03	0.70	− 0.19	0.10	−0.06	0.07

*BMI* body mass index, *eGFR* estimated glomerular filtration rate, *LDL-C* low-density lipoprotein-cholesterol, *SE* standard error

### Association of FEUA with genetic variables

Genotype distribution, call rate, and Hardy-Weinberg equilibrium data are shown in Additional file 1: Table S5. In the Polynesian ancestry groups, no association between *SLC2A9* and FEUA was observed (Table 3). In Eastern Polynesian participants, all had at least one *SLC2A9* effect allele, and in Western Polynesian participants, only one participant did not carry the effect allele (Additional file 1: Table S5). Furthermore, no other SNPs met experiment-wide significance for association with FEUA in any Polynesian ancestry group. In contrast, in New Zealand European participants, association with FEUA was observed at experiment-wide significance for *SLC2A9* (standardised *β* − 0.17, *P* = 2.39 × 10^− 8^), (Table 3). There were 915 (96.8%) New Zealand European participants with genotyping data for *SLC2A9* who had at least one *SLC2A9* effect allele (Additional file 1: Table S5).

**Table 3:** Linear regression analysis of 10 single nucleotide polymorphisms with fractional excretion of uric acid

Gene SNP	Eastern Polynesian *n* = 483					Western Polynesian *n* = 282					New Zealand European *n* = 948				
	Effect allele freq	*β*	SE *β*	Stan. *β*	P	Effect allele freq	*β*	SE *β*	Stan. *β*	*P*	Effect allele freq	*β*	SE *β*	Stan. *β*	*P*
*ABCG2* rs2231142	0.102	0.48	0.29	0.07	0.10	0.302	−0.25	0.21	−0.07	0.24	0.195	−0.06	0.15	−0.01	0.70
*GCKR* rs1260326	0.341	−0.22	0.18	−0.05	0.24	0.266	0.05	0.22	0.01	0.82	0.430	−0.28	0.13	−0.07	0.03
*HLF* rs7224610	0.192	0.34	0.13	0.11	0.09	0.164	0.44	0.13	0.11	0.01	0.429	0.21	0.19	0.06	0.30
*IGF1R* rs6598541	0.798	−0.34	0.24	−0.08	0.16	0.888	0.43	0.41	0.08	0.30	0.380	0.06	0.13	0.02	0.64
*NFAT5* rs7193778	0.119	−0.15	0.18	−0.03	0.40	0.148	−0.15	0.17	−0.03	0.40	0.835	−0.41	0.25	−0.08	0.11
*RREB1* rs675209	0.727	−0.36	0.19	−0.09	0.06	0.885	4.49 × 10^−3^	0.33	0.00	0.99	0.278	−0.09	0.13	0.02	0.51
*SLC22A11* rs2078267	0.840	−0.55	0.23	−0.11	0.02	0.945	0.09	0.47	0.01	0.84	0.535	0.03	0.12	0.01	0.80
*SLC22A12* rs3825018	0.681	−0.21	0.20	−0.05	0.28	0.710	0.07	0.26	0.02	0.79	0.296	−0.24	0.13	−0.06	0.07
*SLC2A9* rs11942223	0.935	−0.80	0.37	−0.10	0.03	0.966	0.86	0.57	0.09	0.13	0.827	−0.86	0.15	−0.17	2.39 × 10^−8^
*UBE2Q2* rs1394125	0.162	−0.17	0.23	−0.03	0.47	0.056	−0.21	0.45	−0.03	0.63	0.623	0.13	0.12	0.03	0.29

Data adjusted for age and sex. *Freq* frequency, *SE* standard error, *SNP* single nucleotide polymorphism, *Stan.* standardised

Association for FEUA at nominal significance (*P* < 0.05) was observed for *SLC22A11* and *SLC2A9* in Eastern Polynesian participants, *HLF* in Western Polynesian participants, and *GCKR* in New Zealand European participants (Table 3). Genotype distribution, call rate, and Hardy-Weinberg equilibrium data for participants analysed according to self-reported ethnicity are shown in Additional file 1: Table S6; no SNPs met experiment-wide significance for association with FEUA in this analysis (Additional file 1: Table S7).

A higher polygenic score was associated with a higher FEUA in all ancestry groups in the univariate analysis (Table 4). In a repeat analysis that excluded *SLC2A9* from the polygenic score modelling, a higher polygenic score was still associated with a higher FEUA in Eastern Polynesian and New Zealand European participants, with a similar trend observed in Western Polynesian participants (Table 4). When analysing according to ethnicity group, a higher polygenic score was also associated with a higher FEUA in Māori (*β* 15.36, SE 3.69, standardised *β* 0.21, *P* = 3.95 × 10^− 5^) and Pacific peoples (*β* 15.08, SE 6.39, standardised *β* 0.12, *P* = 0.02).

**Table 4:** Univariate linear regression analysis of polygenic score with fractional excretion of uric acid

Polygenic score	Eastern Polynesian				Western Polynesian				New Zealand European			
	*β*	SE *β*	Standardised *β*	*P*	*β*	SE *β*	Standardised *β*	*P*	*β*	SE *β*	Standardised *β*	*P*
Including *SLC2A9*	11.71	2.83	0.19	4.11 × 10^−5^	14.63	6.98	0.12	0.04	13.91	2.23	0.20	6.52 × 10^−10^
Not including *SLC2A9*	10.69	2.92	0.16	2.78 × 10^−4^	15.87	8.94	0.11	0.08	11.45	3.88	0.10	3.25 × 10^−3^

A higher polygenic score indicates a greater genetic predisposition for a higher FEUA. *SE* standard error

### Association of FEUA with clinical and genetic variables in linear regression analysis

In both Eastern Polynesian participants and New Zealand European participants, BMI ≥ 30 kg/m^2^, diuretic use, male sex, and polygenic score were independent predictors of FEUA (Table 5). These four variables explained 10% of the FEUA variance in Eastern Polynesian participants, and 22% in New Zealand European participants. For Western Polynesian participants, only the polygenic score was an independent predictor of FEUA, explaining 4% of FEUA variance (Table 5). In Western Polynesian participants, effect sizes for BMI ≥ 30 kg/m^2^ (standardised *β* − 0.13, *P* = 0.053), male sex (standardised *β* − 0.11, *P* = 0.08) and diuretic use (standardised *β* − 0.13, *P* = 0.07) were similar to participants of Eastern Polynesian and New Zealand European ancestry and were approaching statistical significance for predictors of FEUA.

**Table 5:** Predictors of fractional excretion of uric acid in linear regression analysis

	Variable	Standardised *β*	*P*	Model summary
Eastern Polynesian	Body mass index ≥ 30 kg/m^2^	− 0.18	4.58 × 10^− 4^	*R*^2^ = 0.10, *F* = 6.20, *P* = 2.98 × 10^−8^
	Diuretic use	− 0.11	0.03	
	Male sex	− 0.11	0.02	
	Polygenic score*	0.14	0.01	
Western Polynesian	Polygenic score*	0.12	0.049	*R*^2^ = 0.04, *F* = 2.31, *P* = 0.02
New Zealand European	Body mass index ≥ 30 kg/m^2^	− 0.21	3.54 × 10^−10^	*R*^2^ = 0.23, *F* = 28.56, *P* = 1.63 × 10^−43^
	Diuretic use	− 0.12	4.59 × 10^− 4^	
	Male sex	− 0.27	3.03 × 10^−16^	
	Polygenic score*	0.15	1.02 × 10^−6^	

*A higher polygenic score indicates a greater genetic predisposition for a higher FEUA

The same predictors of FEUA were found in all ancestry groups when BMI and eGFR were entered as continuous variables in the models and when serum creatinine was substituted for eGFR (data not shown). Similarly, when alcohol intake was analysed according to the number of grams of alcohol intake per week, the same predictors for FEUA were found in all ancestry groups (data not shown).

Data for the association of clinical variables and the polygenic score with individual components of the FEUA calculation are shown in Additional file 1: Tables S8 and S9. For Eastern Polynesian participants, male sex was associated with all individual components of the FEUA calculation in the multivariable linear regression analysis (Additional file 1: Table S9). BMI ≥ 30 kg/m^2^ was positively associated with serum urate, and diuretic use was positively associated with serum creatinine. For Western Polynesian participants, male sex was also associated with all individual components of the FEUA calculation in the multivariable linear regression analysis. BMI ≥ 30 kg/m^2^ was approaching statistical significance for association with serum urate and serum creatinine. Diuretic use was associated with a higher serum urate and serum creatinine, and the FEUA polygenic score was inversely associated with serum urate. In New Zealand European participants, male sex, BMI ≥ 30 kg/m^2^, diuretic use and polygenic score were associated or approaching statistical significance for association with all components of the FEUA calculation (Additional file 1: Table S9).

When Polynesian participants were analysed according to ethnicity, BMI ≥ 30 kg/m^2^, polygenic score and male sex were independent predictors for FEUA in Māori participants, explaining 11% of FEUA variance (Additional file 1: Table S10). For Pacific peoples, BMI ≥ 30 kg/m^2^ and diuretic use were independent predictors for FEUA and explained 5% of FEUA variance. In this group, polygenic score was approaching statistical significance as a predictor for FEUA (standardised *β* 0.09, *P* = 0.07).

## Discussion

This study has identified independent clinical and genetic variables that contribute to fractional excretion of uric acid. In people of New Zealand European ancestry, these variables include high body mass index, diuretic use, male sex and polygenic score. There is a large unexplained variance in FEUA, particularly in Polynesian people.

Sex-specific differences in the epidemiology and clinical characteristics of hyperuricaemia and gout are well described. Hyperuricaemia and gout prevalence is higher in men compared to women [22], and it is unusual for pre-menopausal women to present with gout in the absence of other risk factors, such as diuretic use or kidney disease [23, 24]. The uricosuric effect of female sex hormones may explain these differences [25, 26]. In the regression models, male sex was associated with a lower FEUA in Eastern Polynesian and New Zealand European participants and was approaching statistical significance for Western Polynesian participants (*P* = 0.08).

Diuretic use was associated with a lower FEUA in Eastern Polynesian and New Zealand European participants, with a similar trend for Western Polynesian participants. The association of diuretic use with hyperuricaemia [27] and gout [28] is well recognised. Diuretics are thought to affect renal uric acid excretion through their action on renal uric acid transporters. Possible mechanisms include diuretic-uric acid exchange via OAT4 on the basolateral membrane of renal tubular cells [29], and inhibition of urate transporters such as OAT1 and OAT3 on the basolateral membrane [5], and MRP4 and NPT4 on the apical membrane [5, 30]. Diuretics also affect renal uric acid excretion via indirect mechanisms related to intravascular volume contraction and salt loss which stimulates renal solute (including uric acid) reabsorption [31].

A high BMI was associated with a lower FEUA in Eastern Polynesian and New Zealand European participants and was approaching statistical significance for Western Polynesian participants. Although BMI is known to negatively correlate with FEUA, it is currently unclear whether this is a direct effect of BMI itself, or associated mediators (such as high circulating insulin, triglycerides, glucose) [3, 32]. Our group has previously reported no association between FEUA and BMI in a fasting state, but that people with overweight/obesity range BMI have a lower FEUA response after hyperuricaemia induced by a fructose load [33].

Of the genetic loci associated with serum urate, the *SLC2A9* variant exerts the greatest effect accounting for approximately 3–4% of inter-individual variance in European people [9]. *SLC2A9* encodes GLUT9, a high capacity urate transporter expressed in proximal renal tubular cells and the liver [34]. The *SLC2A9* variant is more prevalent in Eastern and Western Polynesian people compared to New Zealand European people and confers a strong risk for gout [35]. In our study, all Eastern Polynesian participants had at least one *SLC2A9* effect allele, and in Western Polynesian people, only one participant did not carry the effect allele. This would have eliminated the power to detect associations between FEUA and the *SLC2A9* variant in these ancestry groups. Other potential explanations for a lack of association include undiscovered population-specific genetic variants in *SLC2A9* and in other genes, differences in clinical factors mediating renal uric acid excretion such as insulin resistance, or other factors (genetic or clinical) interacting with *SLC2A9*. For example, a Western Polynesian-specific genetic variant in *ABCC4* associates with FEUA in men [36]. Despite the lack of association with *SLC2A9*, the polygenic score was a predictor for FEUA in Eastern Polynesian and Western Polynesian participants. This suggests that despite the lack of experiment-wide association for a single SNP with FEUA, the cumulative effects of multiple serum urate-associated SNPs can still potentially influence FEUA in these ancestry groups.

The lack of association between *ABCG2* and FEUA confirms the previous finding of Kannangara et al. [37] and is expected due to overlapping datasets. The finding is also notable as it differs from the results of a larger European analysis which demonstrated a small (non-experiment-wide significant) reduction in FEUA with the urate-raising variant [9]. The lack of association in our study is in keeping with the likely mechanism of hyperuricaemia induced by genetic variants of *ABCG2*. The ABCG2 transporter functions predominantly as a gut secretory urate transporter with excretion of urate blocked in the presence of the effect allele. This reduced extra-renal urate clearance results in a ‘renal overload’ hyperuricaemia and a relative increase in the daily excretion of urate without a change in FEUA.

An association between *GCKR* and FEUA was observed in New Zealand Europeans at nominal significance and replicates the findings by Köttgen et al. [9]. GCKR influences the hepatic production of glucose-6-phosphate, which is a precursor for de novo purine synthesis. It also associates with triglyceride [38], and the association with serum urate is attenuated by triglyceride levels [39]. It is unclear how the GCKR protein may influence FEUA. It may be that the genetic variant of *GCKR* influences the levels of a metabolite in the glycolytic pathway that alters renal uric acid excretion.

Our study showed a large unexplained variance in FEUA, particularly in Eastern and Western Polynesian groups. This is despite testing all available clinical variables and the polygenic score in the multivariable linear regression analysis. In the Eastern Polynesian and New Zealand European analysis, the same four variables were found to be predictors for FEUA; however, the overall variance explained was much lower for the Eastern Polynesian group (10% vs 22%). Genetic variants of other loci (that may have population-specific effects) not tested in this analysis may account for some of the unexplained variance. Another explanation is the presence of rare or low-frequency genetic variants associated with serum urate that were not tested in this study. These variants have been shown to exert a larger effect individually on serum urate than the common variants that are mapped into the same loci [40].

This study has a number of strengths and limitations. An important strength is the recruitment of Māori and Pacific peoples, who have a high prevalence of early-onset and severe gout. A further strength was the standardised study visits and methodology. Limitations include the modest sample size for our analysis, particularly for Māori and Pacific participants, which reduced the power to detect associations with FEUA in the Polynesian ancestry groups. Secondly, although our study tested ten FEUA-associated SNPs, it is possible that SNPs associated with serum creatinine may also associate with FEUA and provide a further genetic contribution to the overall FEUA variance. Genotyping data for serum creatinine-associated SNPs were not available for this analysis; however, a positive association between the serum urate-raising SNP of *GCKR* with eGFR and a negative association between the serum urate-raising SNP of *UBE2Q2* with eGFR have been previously reported [41, 42]. Thirdly, as the FEUA-associated genetic variants tested in this study were identified by genome-wide analysis in a European population [9], the influence of these genetic variants cannot be directly generalised to Polynesian ancestry groups. However, we note the consistent pattern of association of the ten variants with gout between European and Polynesian populations [12] (five of the variants were significantly and directionally consistently associated with gout in both Europeans and Polynesians with an average difference in odds ratio of only 0.16), suggesting that some degree of generalisability is possible.

## Conclusion

Both clinical and genetic variables contribute to renal clearance of uric acid*. SLC2A9* rs11942223 exerts effects on FEUA variance in people of European ancestry, but not in Eastern and Western Polynesian people. There is a large unexplained variance in FEUA, particularly in people of Polynesian ancestry.

## Supplementary information

**Additional file 1:** **Table S1.** Power to detect association between single nucleotide polymorphisms and fractional excretion of uric acid. Table S2. Power to detect association between single nucleotide polymorphisms and fractional excretion of uric acid. Table S3. Power to detect association between single nucleotide polymorphisms and fractional excretion of uric acid. Table S4. Power to detect association between single nucleotide polymorphisms and fractional excretion of uric acid. Table S5. Genotype distribution, call rate, and Hardy-Weinberg equilibrium data for fractional excretion of uric acid-associated single nucleotide polymorphisms. Table S6. Genotype distribution, call rate, and Hardy-Weinberg equilibrium data for fractional excretion of uric acid-associated single nucleotide polymorphisms. Table S7. Association analysis of 10 single nucleotide polymorphisms with fractional excretion of uric acid. Table S8. Univariate linear regression analysis of male sex, body mass index, diuretic use and polygenic score with variables included in fractional excretion of uric acid calculation. Table S9. Multivariable linear regression analysis of male sex, body mass index, diuretic use, and polygenic score with clinical variables included in fractional excretion of uric acid calculation. Table S10. Predictors of fractional excretion of uric acid in linear regression analysis. Figure S1. Frequency distribution of fractional excretion of uric acid in all participants and according to ancestry group. FEUA, fractional excretion of uric acid.

## Data Availability

Owing to consent restrictions, the individual-level data used for this study cannot be made publicly available. All data generated or analysed during this study are included in this published article (and its supplementary information files).

## Abbreviations

*ABCC4*: ATP-binding cassette subfamily C member 4
*ABCG2*: ATP-binding cassette subfamily G member 2
ANOVA: Analysis of variance
BMI: Body mass index
eGFR: Estimated glomerular filtration rate
FEUA: Fractional excretion of uric acid
*GCKR*: Glucokinase regulator
GWAS: Genome-wide association study
HLF: Hepatic leukaemia factor
LDL-C: Low-density lipoprotein-cholesterol
SD: Standard deviation
*SLC17A1*: Solute carrier family 17 member 1
*SLC22A11*: Solute carrier family 22 member 11
*SLC22A12*: Solute carrier family 22 member 12
*SLC2A9*: Solute carrier family 2 member 9
SNP: Single nucleotide polymorphism
