# Supplementary material for: Population-specific factors associated with fractional excretion of uric acid
Source: Arthritis Res Ther. 2019 Nov 12;21:234. doi: 10.1186/s13075-019-2016-6 (PMC6852918; doi:10.1186/s13075-019-2016-6)
Supplement: Supplementary file 1 — Additional file 1: Table S1. Power to detect association between single nucleotide polymorphisms and fractional excretion of uric acid. Table S2. Power to detect association between single nucleotide polymorphisms and fractional excretion of uric acid. Table S3. Power to detect association between single nucleotide polymorphisms and fractional excretion of uric acid. Table S4. Power to detect association between single nucleotide polymorphisms and fractional excretion of uric acid. Table S5. Genotype distribution, call rate, and Hardy-Weinberg equilibrium data for fractional excretion of uric acid-associated single nucleotide polymorphisms. Table S6. Genotype distribution, call rate, and Hardy-Weinberg equilibrium data for fractional excretion of uric acid-associated single nucleotide polymorphisms. Table S7. Association analysis of 10 single nucleotide polymorphisms with fractional excretion of uric acid. Table S8. Univariate linear regression analysis of male sex, body mass index, diuretic use and polygenic score with variables included in fractional excretion of uric acid calculation. Table S9. Multivariable linear regression analysis of male sex, body mass index, diuretic use, and polygenic score with clinical variables included in fractional excretion of uric acid calculation. Table S10. Predictors of fractional excretion of uric acid in linear regression analysis. Figure S1. Frequency distribution of fractional excretion of uric acid in all participants and according to ancestry group. FEUA, fractional excretion of uric acid. [file 13075_2019_2016_MOESM1_ESM.zip › Additional File 1_revision.docx]

**Supplementary Table 1.** Power to detect association between single nucleotide polymorphisms and fractional excretion of uric acid.

| ***Gene***  **SNP** | **Effect allele frequency** | | | **FEUA mean (SD), %** | | | **Presumed genetic effect size on FEUA (±%)** | **% Power to detect association at Type 1 error of 0.005** | | |
| --- | --- | --- | --- | --- | --- | --- | --- | --- | --- | --- |
|  | **Eastern Polynesian** | **Western Polynesian** | **New Zealand European** | **Eastern Polynesian** | **Western Polynesian** | **New Zealand European** |  | **Eastern Polynesian** | **Western Polynesian** | **New Zealand European** |
| *ABCG2*  rs2231142 | 0.102 | 0.302 | 0.195 | 5.13 (2.70) | 4.70 (5.89) | 5.89 (2.73) | 0.2 | 1.8 | 2.7 | 6.2 |
|  |  |  |  |  |  |  | 0.4 | 7.9 | 15.0 | 39.2 |
|  |  |  |  |  |  |  | 0.6 | 23.8 | 44.4 | 84.1 |
|  |  |  |  |  |  |  | 0.8 | 49.7 | 77.8 | 98.9 |
|  |  |  |  |  |  |  | 1.0 | 75.8 | 95.4 | >99.9 |
| *SLC22A11*  *rs2078267* | 0.840 | 0.945 | 0.535 |  |  |  | 0.2 | 2.5 | 1.0 | 11.2 |
|  |  |  |  |  |  |  | 0.4 | 13.2 | 2.7 | 64.9 |
|  |  |  |  |  |  |  | 0.6 | 39.5 | 6.8 | 97.7 |
|  |  |  |  |  |  |  | 0.8 | 72.2 | 14.7 | >99.9 |
|  |  |  |  |  |  |  | 1.0 | 92.7 | 27.2 | >99.9 |
| *SLC22A12*  rs3825018 | 0.681 | 0.710 | 0.296 |  |  |  | 0.2 | 4.2 | 2.7 | 8.9 |
|  |  |  |  |  |  |  | 0.4 | 25.6 | 14.5 | 54.5 |
|  |  |  |  |  |  |  | 0.6 | 66.6 | 43.1 | 94.3 |
|  |  |  |  |  |  |  | 0.8 | 93.7 | 76.5 | 99.9 |
|  |  |  |  |  |  |  | 1.0 | 99.6 | 94.9 | >99.9 |
| *SLC2A9*  rs11942223 | 0.718 | 0.966 | 0.827 |  |  |  | 0.2 | 3.8 | 0.8 | 5.5 |
|  |  |  |  |  |  |  | 0.4 | 23.3 | 1.8 | 34.8 |
|  |  |  |  |  |  |  | 0.6 | 62.4 | 3.9 | 80.0 |
|  |  |  |  |  |  |  | 0.8 | 91.5 | 7.9 | 98.0 |
|  |  |  |  |  |  |  | 1.0 | 99.3 | 14.5 | >99.9 |

Power was calculated for a range of presumed effect sizes (0.2-1.0%). FEUA, fractional excretion of uric acid; SD, standard deviation; SNP, single nucleotide polymorphism.

**Supplementary Table 2.** Power to detect association between single nucleotide polymorphisms and fractional excretion of uric acid.

| ***Gene***  **SNP** | **Effect allele frequency** | | **FEUA mean (SD), %** | | **Presumed genetic effect size on FEUA (±%)** | **% Power to detect association at Type 1 error of 0.005** | |
| --- | --- | --- | --- | --- | --- | --- | --- |
|  | **Māori** | **Pacific peoples** | **Māori** | **Pacific peoples** |  | **Māori** | **Pacific peoples** |
| *ABCG2*  rs2231142 | 0.106 | 0.242 | 5.27 (2.68) | 4.68 (2.53) | 0.2 | 1.5 | 3.1 |
|  |  |  |  |  | 0.4 | 6.1 | 18.0 |
|  |  |  |  |  | 0.6 | 18.1 | 51.6 |
|  |  |  |  |  | 0.8 | 39.1 | 84.3 |
|  |  |  |  |  | 1.0 | 64.2 | 97.6 |
| *SLC22A11*  *rs2078267* | 0.818 | 0.940 |  |  | 0.2 | 2.2 | 1.2 |
|  |  |  |  |  | 0.4 | 11.0 | 3.9 |
|  |  |  |  |  | 0.6 | 33.4 | 10.9 |
|  |  |  |  |  | 0.8 | 64.5 | 24.0 |
|  |  |  |  |  | 1.0 | 88.2 | 43.0 |
| *SLC22A12*  rs3825018 | 0.684 | 0.698 |  |  | 0.2 | 3.2 | 3.7 |
|  |  |  |  |  | 0.4 | 18.4 | 21.9 |
|  |  |  |  |  | 0.6 | 52.5 | 59.8 |
|  |  |  |  |  | 0.8 | 85.0 | 90.1 |
|  |  |  |  |  | 1.0 | 97.9 | 99.0 |
| *SLC2A9*  rs11942223 | 0.924 | 0.968 |  |  | 0.2 | 1.2 | 0.9 |
|  |  |  |  |  | 0.4 | 4.3 | 2.1 |
|  |  |  |  |  | 0.6 | 12.0 | 5.0 |
|  |  |  |  |  | 0.8 | 26.4 | 10.5 |
|  |  |  |  |  | 1.0 | 46.7 | 19.4 |

Power was calculated for a range of presumed effect sizes (0.2-1.0%). FEUA, fractional excretion of uric acid; SD, standard deviation; SNP, single nucleotide polymorphism.

**Supplementary Table 3.** Power to detect association between single nucleotide polymorphisms and fractional excretion of uric acid.

| ***Gene***  **SNP** | **Effect allele frequency** | | | **Genetic effect (β) on FEUA *** | | | **% Power to detect association at Type 1 error of 0.005** | | |
| --- | --- | --- | --- | --- | --- | --- | --- | --- | --- |
|  | **Eastern Polynesian** | **Western Polynesian** | **New Zealand European** | **Eastern Polynesian** | **Western Polynesian** | **New Zealand European** | **Eastern Polynesian** | **Western Polynesian** | **New Zealand European** |
| *ABCG2*  rs2231142 | 0.102 | 0.302 | 0.195 | 0.48 | -0.25 | -0.06 | 12.9 | 4.4 | 0.8 |
| *GCKR*  rs1260326 | 0.341 | 0.266 | 0.430 | -0.22 | 0.05 | -0.28 | 5.4 | 0.6 | 27.7 |
| *HLF*  rs7224610 | 0.192 | 0.164 | 0.429 | 0.34 | 0.44 | 0.21 | 10.3 | 10.8 | 12.6 |
| *IGF1R*  rs6598541 | 0.798 | 0.888 | 0.380 | -0.34 | 0.43 | 0.06 | 10.9 | 6.7 | 1.0 |
| *NFAT5*  rs7193778 | 0.119 | 0.148 | 0.835 | -0.15 | -0.15 | -0.41 | 1.3 | 1.1 | 35.4 |
| *RREB1*  rs675209 | 0.727 | 0.885 | 0.278 | -0.36 | 4.49x10^-3^ | -0.09 | 16.9 | 0.5 | 1.6 |
| *SLC22A11*  rs2078267 | 0.840 | 0.945 | 0.535 | -0.55 | 0.09 | 0.03 | 31.6 | 0.6 | 0.6 |
| *SLC22A12*  rs3825018 | 0.681 | 0.71 | 0.296 | -0.21 | 0.07 | -0.24 | 4.7 | 0.7 | 14.5 |
| *SLC2A9*  rs11942223 | 0.935 | 0.966 | 0.827 | -0.80 | 0.86 | -0.86 | 18.3 | 9.6 | 99.2 |
| *UBE2Q2*  rs1394125 | 0.162 | 0.056 | 0.623 | -0.17 | -0.21 | 0.13 | 1.9 | 1.0 | 3.6 |

* Power was calculated *post hoc* based on effect sizes determined in this analysis. FEUA, fractional excretion of uric acid; SNP, single nucleotide polymorphism.

**Supplementary Table 4.** Power to detect association between single nucleotide polymorphisms and fractional excretion of uric acid.

| ***Gene***  **SNP** | **Effect allele frequency** | | **Genetic effect (β) on FEUA *** | | **% Power to detect association at Type 1 error of 0.005** | |
| --- | --- | --- | --- | --- | --- | --- |
|  | **Māori** | **Pacific peoples** | **Māori** | **Pacific peoples** | **Māori** | **Pacific peoples** |
| *ABCG2*  rs2231142 | 0.106 | 0.242 | 0.33 | -0.12 | 3.9 | 1.3 |
| *GCKR*  rs1260326 | 0.371 | 0.259 | -0.53 | 0.22 | 43.1 | 4.1 |
| *HLF*  rs7224610 | 0.204 | 0.164 | -0.40 | 0.74 | 12.4 | 59.2 |
| *IGF1R*  rs6598541 | 0.749 | 0.901 | -0.36 | 0.40 | 11.4 | 6.8 |
| *NFAT5*  rs7193778 | 0.130 | 0.129 | -0.35 | 0.17 | 5.5 | 1.5 |
| *RREB1*  rs675209 | 0.679 | 0.887 | -0.26 | -0.16 | 5.9 | 1.3 |
| *SLC22A11*  rs2078267 | 0.818 | 0.940 | -0.43 | -0.34 | 13.4 | 2.8 |
| *SLC22A12*  rs3825018 | 0.684 | 0.698 | -0.01 | -0.25 | 0.5 | 6.2 |
| *SLC2A9*  rs11942223 | 0.924 | 0.968 | -0.62 | 0.33 | 13.1 | 1.5 |
| *UBE2Q2*  rs1394125 | 0.179 | 0.069 | -0.16 | -0.31 | 1.5 | 2.6 |

* Power was calculated *post hoc* based on effect sizes determined in this analysis. FEUA, fractional excretion of uric acid; SNP, single nucleotide polymorphism.

**Supplementary Table 5.** Genotype distribution, call rate, and Hardy-Weinberg equilibrium data for fractional excretion of uric acid-associated single nucleotide polymorphisms.

| ***Gene***  **SNP** | **Effect allele** | **Genotype** | **Eastern Polynesian**  **n=483** | | | **Western Polynesian**  **n=282** | | | **New Zealand European**  **n=948** | | |
| --- | --- | --- | --- | --- | --- | --- | --- | --- | --- | --- | --- |
|  |  |  | **Genotype**  **n (%)** | **Call rate (%)** | **HWE Chi-square*** | **Genotype**  **n (%)** | **Call rate (%)** | **HWE Chi-square*** | **Genotype**  **n (%)** | **Call rate (%)** | **HWE Chi-square*** |
| *ABCG2*  rs2231142 | T | Missing | 9 (1.9%) | 98.1 | 0.330 | 9 (3.2%) | 96.8 | 3.963 | 28 (3.0%) | 97.0 | 0.991 |
|  |  | GG | 380 (78.7%) |  |  | 146 (51.8%) |  |  | 606 (63.9%) |  |  |
|  |  | TG | 91 (18.8%) |  |  | 89 (31.6%) |  |  | 270 (28.5%) |  |  |
|  |  | TT | 3 (0.6%) |  |  | 38 (13.5%) |  |  | 44 (4.6%) |  |  |
| *GCKR*  rs1260326 | T | Missing | 29 (6.0%) | 94.0 | 0.005 | 21 (7.4%) | 92.6 | 6.042 | 116 (12.2%) | 87.8 | 1.314 |
|  |  | CC | 197 (40.8%) |  |  | 149 (52.8%) |  |  | 274 (28.9%) |  |  |
|  |  | TC | 204 (42.2%) |  |  | 85 (30.1%) |  |  | 401 (42.3%) |  |  |
|  |  | TT | 53 (11.0%) |  |  | 27 (9.6%) |  |  | 157 (16.6%) |  |  |
| *HLF*  rs7224610 | C | Missing | 163 (33.7%) | 66.3 | 1.131 | 99 (35.1%) | 64.9 | 0.001 | 540 (57.0%) | 43.0 | 0.024 |
|  |  | AA | 211 (43.7%) |  |  | 128 (45.4%) |  |  | 135 (14.2%) |  |  |
|  |  | CA | 95 (19.7%) |  |  | 50 (17.7%) |  |  | 196 (20.7%) |  |  |
|  |  | CC | 14 (2.9%) |  |  | 5 (1.8%) |  |  | 77 (8.1%) |  |  |
| *IGF1R*  rs6598541 | A | Missing | 163 (33.7%) | 66.3 | 1.411 | 99 (35.1%) | 64.9 | 0.636 | 116 (12.2%) | 87.8 | 0.098 |
|  |  | GG | 19 (3.9%) |  |  | 3 (1.1%) |  |  | 319 (33.6%) |  |  |
|  |  | AG | 91 (18.8%) |  |  | 35 (12.4%) |  |  | 393 (41.5%) |  |  |
|  |  | AA | 210 (43.5%) |  |  | 145 (51.4%) |  |  | 120 (12.7%) |  |  |
| *NFAT5*  rs7193778 | C | Missing | 164 (34.0%) | 66.0 | 0.059 | 99 (35.1%) | 64.9 | 0.018 | 540 (57.0%) | 43.0 | 2.534 |
|  |  | TT | 247 (51.1%) |  |  | 134 (47.5%) |  |  | 10 (1.1%) |  |  |
|  |  | CT | 68 (14.1%) |  |  | 44 (15.6%) |  |  | 115 (12.1%) |  |  |
|  |  | CC | 4 (0.8%) |  |  | 5 (1.8%) |  |  | 283 (29.9%) |  |  |
| *RREB1*  rs675209 | T | Missing | 29 (6.0%) | 94.0 | 2.263 | 22 (7.8%) | 92.2 | 0.447 | 81 (8.5%) | 91.5 | 0.476 |
|  |  | CC | 41 (8.5%) |  |  | 4 (1.4%) |  |  | 463 (48.8%) |  |  |
|  |  | TC | 166 (34.4%) |  |  | 52 (18.4%) |  |  | 326 (34.4%) |  |  |
|  |  | TT | 247 (51.1%) |  |  | 204 (72.3%) |  |  | 78 (8.2%) |  |  |
| *SLC22A11*  rs2078267 | C | Missing | 23 (4.8%) | 95.2 | 0.043 | 19 (6.7%) | 93.3 | 0.498 | 47 (5.0%) | 95.0 | 0.013 |
|  |  | TT | 14 (2.9%) |  |  | 1 (0.4%) |  |  | 194 (20.5%) |  |  |
|  |  | CT | 119 (24.6%) |  |  | 25 (8.9%) |  |  | 450 (47.5%) |  |  |
|  |  | CC | 327 (67.7%) |  |  | 237 (84.0%) |  |  | 257 (27.1%) |  |  |
| *SLC22A12*  rs3825018 | A | Missing | 73 (15.1%) | 84.9 | 2.308 | 61 (21.6%) | 78.4 | 0.656 | 76 (8.0%) | 92.0 | 3.532 |
|  |  | GG | 35 (7.2%) |  |  | 15 (5.3%) |  |  | 432 (45.6%) |  |  |
|  |  | AG | 192 39.8%) |  |  | 98 (34.8%) |  |  | 363 (38.3%) |  |  |
|  |  | AA | 183 (37.9%) |  |  | 108 (38.3%) |  |  | 77 (8.1%) |  |  |
| *SLC2A9*  rs11942223 | T | Missing | 22 (4.6%) | 95.4 | 1.609 | 18 (6.4%) | 93.6 | 3.600 | 3 (0.3%) | 99.7 | 0.208 |
|  |  | CC | 0 (0.0%) |  |  | 1 (0.4%) |  |  | 30 (3.2%) |  |  |
|  |  | TC | 60 (12.4%) |  |  | 16 (5.7%) |  |  | 268 (28.3%) |  |  |
|  |  | TT | 401 (83.0%) |  |  | 247 (87.6%) |  |  | 647 (68.2%) |  |  |
| *UBE2Q2*  rs1394125 | A | Missing | 29 (6.0%) | 94.0 | 0.003 | 21 (7.4%) | 92.6 | 2.282 | 83 (8.8%) | 91.2 | 6.706 |
|  |  | GG | 322 (66.7%) |  |  | 234 (83.0%) |  |  | 139 (14.7%) |  |  |
|  |  | AG | 117 (24.2%) |  |  | 25 (8.9%) |  |  | 374 (39.5%) |  |  |
|  |  | AA | 15 (3.1%) |  |  | 2 (0.7%) |  |  | 352 (37.1%) |  |  |

*Hardy-Weinberg equilibrium data for participants without gout. HWE, Hardy-Weinberg equilibrium; SNP, single nucleotide polymorphism.

**Supplementary Table 6.** Genotype distribution, call rate, and Hardy-Weinberg equilibrium data for fractional excretion of uric acid-associated single nucleotide polymorphisms.

| ***Gene***  **SNP** | **Effect allele** | **Genotype** | **Māori**  **n=376** | | | **Pacific peoples**  **n=389** | | |
| --- | --- | --- | --- | --- | --- | --- | --- | --- |
|  |  |  | **Genotype**  **n (%)** | **Call rate (%)** | **HWE Chi-square*** | **Genotype**  **n (%)** | **Call rate (%)** | **HWE Chi-square*** |
| *ABCG2*  rs2231142 | T | Missing | 9 (2.4%) | 97.6 | 0.114 | 9 (2.3%) | 97.9 | 4.330 |
|  |  | GG | 291 (77.4%) |  |  | 235 (60.4%) |  |  |
|  |  | TG | 74 (19.7%) |  |  | 106 (27.2%) |  |  |
|  |  | TT | 2 (0.5%) |  |  | 39 (10.0%) |  |  |
| *GCKR*  rs1260326 | T | Missing | 24 (6.4%) | 93.6 | 0.183 | 26 (6.7%) | 93.3 | 5.367 |
|  |  | CC | 138 (36.7%) |  |  | 208 (53.5%) |  |  |
|  |  | TC | 167 (44.4%) |  |  | 122 (31.4%) |  |  |
|  |  | TT | 47 (12.5%) |  |  | 33 (8.5%) |  |  |
| *HLF*  rs7224610 | C | Missing | 145 (38.6%) | 61.4 | 0.690 | 117 (30.1%) | 59.9 | 0.122 |
|  |  | AA | 149 (39.6%) |  |  | 190 (48.8%) |  |  |
|  |  | CA | 70 (18.6%) |  |  | 75 (19.3%) |  |  |
|  |  | CC | 12 (3.2%) |  |  | 7 (1.8%) |  |  |
| *IGF1R*  rs6598541 | A | Missing | 145 (38.6%) | 61.4 | 0.430 | 117 (30.1%) | 59.9 | 0.229 |
|  |  | GG | 19 (5.1%) |  |  | 3 (0.8%) |  |  |
|  |  | AG | 78 (20.7%) |  |  | 48 (12.3%) |  |  |
|  |  | AA | 134 (35.6%) |  |  | 221 (56.8%) |  |  |
| *NFAT5*  rs7193778 | C | Missing | 146 (38.8%) | 61.2 | 0.226 | 117 (30.1%) | 59.9 | 0.001 |
|  |  | TT | 174 (46.3%) |  |  | 207 (53.2%) |  |  |
|  |  | CT | 52 (13.8%) |  |  | 60 (15.4%) |  |  |
|  |  | CC | 4 (1.1%) |  |  | 5 (1.3%) |  |  |
| *RREB1*  rs675209 | T | Missing | 24 (6.4%) | 93.6 | 0.309 | 27 (6.9%) | 93.1 | 0.907 |
|  |  | CC | 40 (10.6%) |  |  | 5 (1.3%) |  |  |
|  |  | TC | 146 (38.8%) |  |  | 72 (18.5%) |  |  |
|  |  | TT | 166 (44.1%) |  |  | 285 (73.3%) |  |  |
| *SLC22A11*  rs2078267 | C | Missing | 19 (5.1%) | 94.9 | 0.159 | 23 (5.9%) | 94.1 | 0.879 |
|  |  | TT | 13 (3.5%) |  |  | 2 (0.5%) |  |  |
|  |  | CT | 104 (27.7%) |  |  | 40 (10.3%) |  |  |
|  |  | CC | 240 (63.8%) |  |  | 324 (83.3%) |  |  |
| *SLC22A12*  rs3825018 | A | Missing | 69 (18.4%) | 91.6 | 0.006 | 65 (16.7%) | 83.3 | 5.312 |
|  |  | GG | 30 (8.0%) |  |  | 20 (5.1%) |  |  |
|  |  | AG | 134 (35.6%) |  |  | 156 (40.1%) |  |  |
|  |  | AA | 143 (38.0%) |  |  | 148 (38.0%) |  |  |
| *SLC2A9*  rs11942223 | T | Missing | 22 (5.9%) | 94.1 | 1.716 | 18 (4.6%) | 95.4 | 2.288 |
|  |  | CC | 0 (0.0%) |  |  | 1 (0.3%) |  |  |
|  |  | TC | 54 (14.4%) |  |  | 22 (5.7%) |  |  |
|  |  | TT | 300 (79.8%) |  |  | 348 (89.5%) |  |  |
| *UBE2Q2*  rs1394125 | A | Missing | 24 (6.4%) | 93.6 | 0.001 | 26 (6.7%) | 93.3 | 0.489 |
|  |  | GG | 239 (63.6%) |  |  | 317 (81.5%) |  |  |
|  |  | AG | 100 (26.6%) |  |  | 42 (10.8%) |  |  |
|  |  | AA | 13 (3.5%) |  |  | 4 (1.0%) |  |  |

*Hardy-Weinberg equilibrium data for participants without gout. HWE, Hardy-Weinberg equilibrium; SNP, single nucleotide polymorphism.

**Supplementary Table 7.** Association analysis of 10 single nucleotide polymorphisms with fractional excretion of uric acid.

| **Gene**  **SNP** | **Māori**  **n=376** | | | | | **Pacific peoples**  **n=389** | | | | |
| --- | --- | --- | --- | --- | --- | --- | --- | --- | --- | --- |
|  | **Effect allele freq** | **β** | **SE β** | **Standardised β** | **P** | **Effect allele freq** | **β** | **SE β** | **Standardised β** | **P** |
| *ABCG2*  rs2231142 | 0.106 | 0.33 | 0.33 | 0.05 | 0.31 | 0.242 | -0.12 | 0.20 | -0.03 | 0.53 |
| *GCKR*  rs1260326 | 0.371 | -0.53 | 0.20 | -0.14 | 0.01 | 0.259 | 0.22 | 0.20 | 0.05 | 0.27 |
| *HLF*  rs7224610 | 0.204 | -0.40 | 0.28 | -0.10 | 0.15 | 0.164 | 0.74 | 0.30 | 0.15 | 0.01 |
| *IGF1R*  rs6598541 | 0.749 | -0.36 | 0.25 | -0.09 | 0.15 | 0.901 | 0.40 | 0.37 | 0.07 | 0.28 |
| *NFAT5*  rs7193778 | 0.130 | -0.35 | 0.34 | -0.07 | 0.30 | 0.129 | 0.17 | 0.33 | 0.03 | 0.60 |
| *RREB1*  rs675209 | 0.679 | -0.26 | 0.20 | -0.07 | 0.20 | 0.887 | -0.16 | 0.29 | -0.03 | 0.59 |
| *SLC22A11*  rs2078267 | 0.818 | -0.43 | 0.25 | -0.09 | 0.08 | 0.940 | -0.34 | 0.38 | -0.05 | 0.38 |
| *SLC22A12*  rs3825018 | 0.684 | -0.01 | 0.21 | -3.91x10^-3^ | 0.95 | 0.698 | -0.25 | 0.23 | -0.06 | 0.28 |
| *SLC2A9*  rs11942223 | 0.924 | -0.62 | 0.39 | -0.08 | 0.11 | 0.968 | 0.33 | 0.52 | 0.03 | 0.52 |
| *UBE2Q2*  rs1394125 | 0.179 | -0.16 | 0.25 | -0.03 | 0.53 | 0.069 | -0.31 | 0.35 | -0.05 | 0.37 |

Data adjusted for age and sex. Freq, frequency; SE, standard error; SNP, single nucleotide polymorphism.

**Supplementary Table 8.** Univariate linear regression analysis of male sex, body mass index, diuretic use and polygenic score with variables included in fractional excretion of uric acid calculation.

| **Serum urate** | | | | | | | | | | | | |
| --- | --- | --- | --- | --- | --- | --- | --- | --- | --- | --- | --- | --- |
|  | **Eastern Polynesian** | | | | **Western Polynesian** | | | | **New Zealand European** | | | |
|  | **β** | **SE β** | **Standardised β** | **P** | **β** | **SE β** | **Standardised β** | **P** | **β** | **SE β** | **Standardised β** | **P** |
| Male sex | 0.05 | 0.01 | 0.26 | 1.35x10^-8^ | 0.06 | 0.01 | 0.27 | 5.69x10^-8^ | 0.07 | 0.01 | 0.26 | 7.40x10^-16^ |
| BMI ≥ 30kg/m^2^ | 0.03 | 0.01 | 0.24 | 7.26x10^-8^ | 0.03 | 0.01 | 0.19 | 1.24x10^-3^ | 0.04 | 0.01 | 0.26 | 1.93x10^-15^ |
| Diuretic use | 0.02 | 0.01 | 0.10 | 0.04 | 0.07 | 0.02 | 0.21 | 3.81x10^-4^ | 0.09 | 0.01 | 0.32 | 2.7x10^-23^ |
| Polygenic score | -0.26 | 0.10 | -0.12 | 0.01 | -0.72 | 0.32 | -0.13 | 0.03 | -0.34 | 0.10 | -0.12 | 4.24x10^-4^ |
| **Serum creatinine** | | | | | | | | | | | | |
|  | **Eastern Polynesian** | | | | **Western Polynesian** | | | | **New Zealand European** | | | |
|  | **β** | **SE β** | **Standardised β** | **P** | **β** | **SE β** | **Standardised β** | **P** | **β** | **SE β** | **Standardised β** | **P** |
| Male sex | 20.53 | 2.52 | 0.35 | 3.63x10^-15^ | 28.74 | 3.80 | 0.42 | 5.62x10^-13^ | 18.80 | 2.71 | 0.22 | 7.13x10^-12^ |
| BMI ≥ 30kg/m^2^ | 5.42 | 1.82 | 0.13 | 3.03x10^-3^ | 3.50 | 3.26 | 0.06 | 0.28 | 4.97 | 1.42 | 0.11 | 4.96x10^-4^ |
| Diuretic use | 23.29 | 3.18 | 0.32 | 1.07x10^-12^ | 43.04 | 5.34 | 0.44 | 2.24x10^-14^ | 27.06 | 2.60 | 0.32 | 5.12x10^-24^ |
| Polygenic score | -35.61 | 30.97 | -0.05 | 0.25 | -45.99 | 98.05 | -0.03 | 0.64 | 1.94 | 28.80 | 2.22x10^-3^ | 0.95 |
| **Urine urate/urine creatinine** | | | | | | | | | | | | |
|  | **Eastern Polynesian** | | | | **Western Polynesian** | | | | **New Zealand European** | | | |
|  | **β** | **SE β** | **Standardised β** | **P** | **β** | **SE β** | **Standardised β** | **P** | **β** | **SE β** | **Standardised β** | **P** |
| Male sex | -0.05 | 0.01 | -0.21 | 5.39x10^-6^ | -0.06 | 0.01 | -0.26 | 9.69x10^-6^ | -0.06 | 0.01 | -0.23 | 3.80x10^-13^ |
| BMI ≥ 30kg/m^2^ | -0.02 | 0.01 | -0.13 | 3.67x10^-3^ | -0.01 | 0.01 | -0.04 | 0.45 | -0.03 | 4.18x10^-3^ | -0.20 | 6.09x10^-10^ |
| Diuretic use | -0.06 | 0.01 | -0.19 | 2.41x10^-5^ | -0.06 | 0.02 | -0.19 | 1.07x10^-3^ | -0.04 | 0.01 | -0.18 | 5.19x10^-8^ |
| Polygenic score | 0.30 | 0.12 | 0.11 | 0.01 | 0.47 | 0.30 | 0.09 | 0.12 | 0.25 | 0.08 | 0.10 | 3.37x10^-3^ |

**Supplementary Table 9.** Multivariable linear regression analysis of male sex, body mass index, diuretic use, and polygenic score with clinical variables included in fractional excretion of uric acid calculation.

| **Serum urate** | | | | | | | | | | | | | |
| --- | --- | --- | --- | --- | --- | --- | --- | --- | --- | --- | --- | --- | --- |
|  | **Eastern Polynesian** | | | | **Western Polynesian** | | | | **New Zealand European** | | | |  |
|  | **β** | **SE β** | **Standardised β** | **P** | **β** | **SE β** | **Standardised β** | **P** | **β** | **SE β** | **Standardised β** | **P** |  |
| Male sex | 0.05 | 0.01 | 0.25 | 7.01x10^-8^ | 0.06 | 0.01 | 0.25 | 6.45x10^-5^ | 0.06 | 0.01 | 0.22 | 2.63x10^-12^ |  |
| BMI ≥ 30kg/m^2^ | 0.02 | 0.01 | 0.12 | 0.01 | 0.02 | 0.01 | 0.11 | 0.09 | 0.02 | 4.61x10^-3^ | 0.11 | 6.82x10^-4^ |  |
| Diuretic use | 0.02 | 0.01 | 0.09 | 0.06 | 0.05 | 0.02 | 0.16 | 0.02 | 0.05 | 0.01 | 0.16 | 5.69x10^-7^ |  |
| Polygenic score | -0.13 | 0.1 | -0.06 | 0.19 | -0.72 | 0.31 | -0.13 | 0.02 | -0.19 | 0.08 | -0.06 | 0.03 |  |
| **Serum creatinine** | | | | | | | | | | | | | |
|  | **Eastern Polynesian** | | | | **Western Polynesian** | | | | **New Zealand European** | | | |  |
|  | **β** | **SE β** | **Standardised β** | **P** | **β** | **SE β** | **Standardised β** | **P** | **β** | **SE β** | **Standardised β** | **P** |  |
| Male sex | 21.85 | 1.81 | 0.38 | 4.81x10^-29^ | 26.96 | 2.87 | 0.39 | 3.73x10^-18^ | 18.56 | 1.95 | 0.22 | 1.87x10^-20^ |  |
| BMI ≥ 30kg/m^2^ | -0.33 | 1.34 | -0.01 | 0.81 | -4.38 | 2.37 | -0.08 | 0.07 | -2.28 | 1.04 | -0.05 | 0.03 |  |
| Diuretic use | 6.84 | 2.42 | 0.10 | 0.01 | 17.71 | 4.73 | 0.18 | 2.26x10^-4^ | 6.23 | 2.06 | 0.07 | 2.59x10^-3^ |  |
| Polygenic score | -8.21 | 20.76 | -0.01 | 0.69 | 20.37 | 63.82 | 0.01 | 0.75 | 31.90 | 19.02 | 0.04 | 0.09 |  |
| **Urine urate/urine creatinine** | | | | | | | | | | | | | |
|  | **Eastern Polynesian** | | | | **Western Polynesian** | | | | **New Zealand European** | | | |  |
|  | **β** | **SE β** | **Standardised β** | **P** | **β** | **SE β** | **Standardised β** | **P** | **β** | **SE β** | **Standardised β** | **P** |  |
| Male sex | -0.04 | 0.01 | -0.19 | 3.56x10^-5^ | -0.05 | 0.01 | -0.23 | 2.55x10^-4^ | -0.06 | 0.01 | -0.23 | 8.95x10^-12^ |  |
| BMI ≥ 30kg/m^2^ | -0.01 | 0.01 | -0.09 | 0.09 | 3.08x10^-3^ | 0.01 | 0.02 | 0.78 | -0.01 | 0.04 | -0.10 | 0.01 |  |
| Diuretic use | -0.03 | 0.01 | -0.09 | 0.06 | -0.02 | 0.02 | -0.08 | 0.27 | -0.01 | 0.01 | -0.05 | 0.11 |  |
| Polygenic score | 0.21 | 0.12 | 0.08 | 0.07 | 0.41 | 0.30 | 0.08 | 0.17 | 0.15 | 0.08 | 0.06 | 0.07 |  |

**Supplementary Table 10.** Predictors of fractional excretion of uric acid in linear regression analysis.

| **Māori** | **Variable** | **Standardised β** | **P** | **Model summary** |
| --- | --- | --- | --- | --- |
|  | Body mass index ≥ 30kg/m^2^ | -0.18 | 3.94x10^-3^ | R^2^=0.11, F=5.92, P=1.10x10^-7^ |
|  | Male sex | -0.14 | 0.01 |  |
|  | Polygenic score* | 0.17 | 1.05x10^-3^ |  |
|  | | | | |
| **Pacific peoples** | Body mass index ≥ 30kg/m^2^ | -0.14 | 0.01 | R^2^=0.05, F=2.97, P=2.06x10^-3^ |
|  | Diuretic use | -0.17 | 3.65x10^-3^ |  |

*A higher polygenic score indicates a greater genetic predisposition for a higher FEUA.

**Supplementary Figure Legends**

**Supplementary Figure 1.** Frequency distribution of fractional excretion of uric acid in all participants and according to ancestry group. FEUA, fractional excretion of uric acid.
